# Tuning gradient boosting for imbalanced bioassay modelling with custom loss functions

**DOI:** 10.1186/s13321-022-00657-w

**Published:** 2022-11-10

**Authors:** Davide Boldini, Lukas Friedrich, Daniel Kuhn, Stephan A. Sieber

**Affiliations:** 1 Center for Functional Protein Assemblies, Technical University of Munich (TUM), Ernst-Otto-Fischer-Straße 8, 85784 Garching, Germany; 2 Merck Healthcare KGaA, Frankfurter Straße 250, 64293 Darmstadt, Germany

**Keywords:** Virtual screening, Imbalanced classification, Gradient boosting

## Abstract

While in the last years there has been a dramatic increase in the number of available bioassay datasets, many of them suffer from extremely imbalanced distribution between active and inactive compounds. Thus, there is an urgent need for novel approaches to tackle class imbalance in drug discovery. Inspired by recent advances in computer vision, we investigated a panel of alternative loss functions for imbalanced classification in the context of Gradient Boosting and benchmarked them on six datasets from public and proprietary sources, for a total of 42 tasks and 2 million compounds. Our findings show that with these modifications, we achieve statistically significant improvements over the conventional cross-entropy loss function on five out of six datasets. Furthermore, by employing these bespoke loss functions we are able to push Gradient Boosting to match or outperform a wide variety of previously reported classifiers and neural networks. We also investigate the impact of changing the loss function on training time and find that it increases convergence speed up to 8 times faster. As such, these results show that tuning the loss function for Gradient Boosting is a straightforward and computationally efficient method to achieve state-of-the-art performance on imbalanced bioassay datasets without compromising on interpretability and scalability.

**Graphical Abstract:**

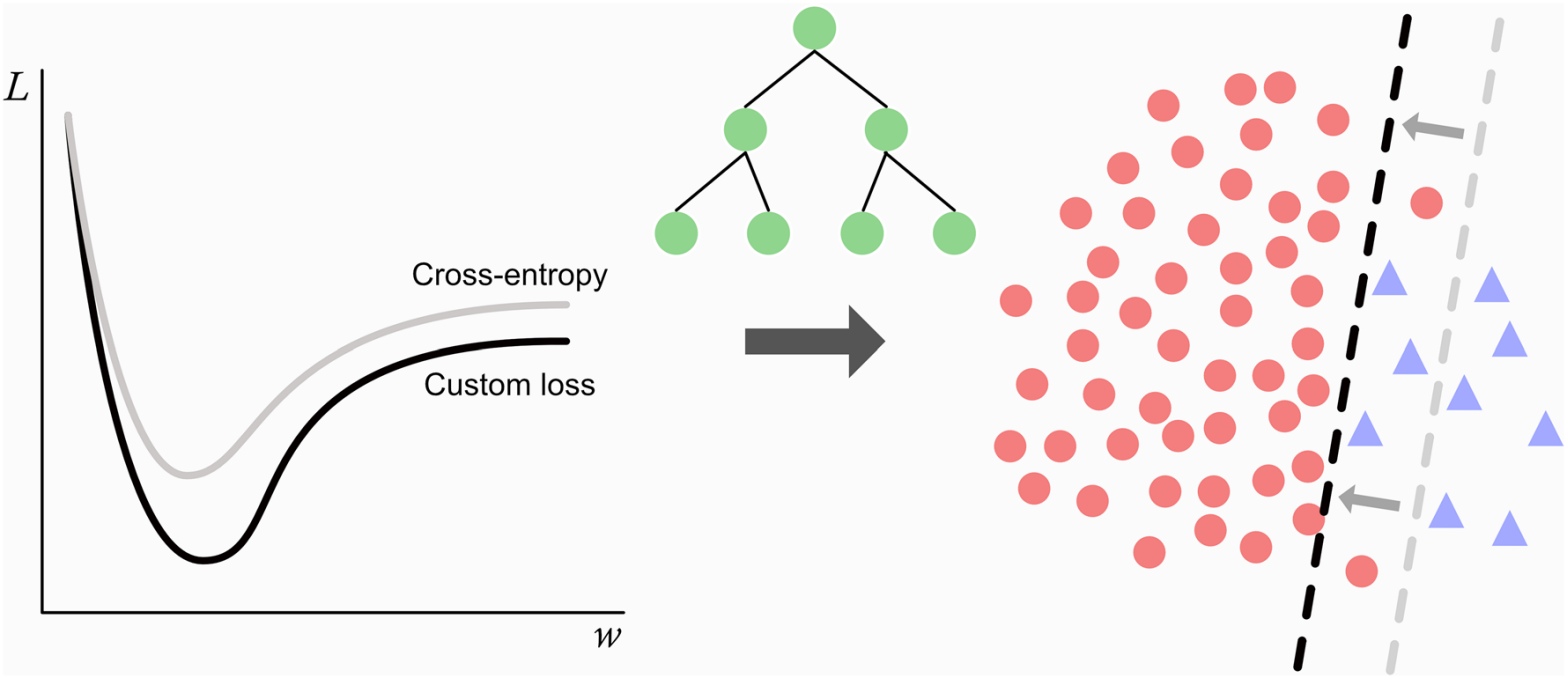

**Supplementary Information:**

The online version contains supplementary material available at 10.1186/s13321-022-00657-w.

## Introduction

In the last decade, machine learning (ML) and deep learning (DL) have radically transformed the conventional workflow for virtual screening in drug discovery [1]. This paradigm shift is strongly related to the substantial increase in freely available chemical data [2]. For example, popular repositories like PubChem and ZINC20 currently contain 1.2 million bioactivity assays and 1.4 billion unique compounds respectively [3–5]. Thanks to these resources, it is straightforward to obtain thousands of training points to develop high-performing predictive models, which can then be used to screen for novel ligands, antibiotics, antivirals and so forth [6–8].

The amount of data available has made it possible to use large neural networks, such as autoencoders (AE), transformers and graph neural networks (GNN) to learn data-driven molecular features, in contrast to prior featurization methods such as fingerprints and physicochemical descriptors [9–11]. Although these architectures have achieved impressive results on many benchmarks, they tend to be outperformed by descriptor-based models on class-imbalanced datasets [12–15], where the number of inactive compounds can be several orders of magnitude larger than the number of actives. Among the descriptor-based classifiers, tree ensembles such as Random Forest and, more recently, Gradient Boosting generally achieve the best performance [13, 15, 16]. Furthermore, this class of models provides additional benefits such as straightforward interpretability [17, 18], fewer hyperparameters to optimize and faster training speed compared to neural networks. [19]

The issue of class imbalance is of critical importance in drug discovery, given that the vast majority of the datasets available in this field are imbalanced [20], as highlighted by Landrum et al. [21] As such, there is an urgent need for novel strategies to tackle class imbalance for modelling bioassay data.

Current methods to address this issue usually rely on resampling the original class distribution or by employing algorithmic solutions such as custom loss functions [22, 23]. The latter approach has garnered interest in the field of computer vision, where the majority of classes in multitask classification have only a handful of positive samples [24–27]. Overall, these approaches rely on reframing the classification objective by reducing the influence of well-classified training instances, forcing the classifier to focus on hard-to-model samples, or by adjusting the unscaled output logits according to the prior probability to observe a given class. Research has shown that employing these methods provides a significant improvement over the baseline with virtually no additional computational cost. [24–27]

While there are several studies investigating resampling in the context of bioassay modelling [5, 28–30], changing the training objective has not been thoroughly investigated thus far. This study directly addresses this gap by investigating the effectiveness of a variety of recently published imbalance-insensitive loss functions for training Gradient Boosting classifiers. In this work, we considered Focal loss (FC) [24] Logit-adjusted loss (LA) [27] Equalization loss (EQ) [26] and Label-Distribution-Aware Margin (LDAM) [25] loss because of their popularity in computer vision and their diversity from a theoretical standpoint.

The choice of pairing Gradient Boosting with the loss functions is motivated by its strong baseline performance across several studies in imbalanced classification tasks [13, 15]. Furthermore, its training speed makes [31, 32] it an attractive solution for modelling large-scale bioassays and its straightforward explicability allows detection of spurious correlations arising from false positives [33], which are known to be frequent in high-throughput screens [34, 35]. Therefore, tuning Gradient Boosting with bespoke loss functions can result in cheap, interpretable and high-performing models which is ideally suited for modelling imbalanced bioassay data.

We benchmark our proposed approach on six datasets from public (MoleculeNet [15] and MolData [20]) and proprietary (Merck KGaA) sources, comprising of approximatively 2 million compounds and 42 tasks with varying degrees of imbalance. Our findings show that changing the loss function provides a consistent, significant improvement, over cross entropy loss on five out of six datasets and that thanks to this modification, Gradient Boosting is able to match or outperform a wide variety of ML and DL approaches, including multitasking networks.

## Methods

### Gradient boosting

Originally developed by Friedman et al. [36] Gradient Boosting is a tree ensemble method that relies on training a sequence of weak learners (generally regression trees), each fitted on the residuals of the prior model. The final model is obtained by simply combining all the predictions from each individual classifier. Since this procedure is prone to overfitting, all Gradient Boosting frameworks offer a variety of regularization options, such as learning rates to modulate the influence of an individual learner on the final prediction, sampling of training samples and variables, L1 regularization and other options. [31, 32]

A key difference between Gradient Boosting and Random Forest is in the way individual trees are optimized. A Gradient Boosting classifier uses regression trees, where the individual splits are optimized according to the gradient and the Hessian of some loss function (i.e. cross-entropy), and converts the sum of predictions into a probability by applying the sigmoid function [31]. Random Forest instead uses decision trees, where the individual splits are optimized using criteria such as the Gini impurity or the Shannon entropy [37]. This distinction allows implementation of custom loss functions in a straightforward manner in any Gradient Boosting framework. [38]

There are several python packages available for training Gradient Boosting models, the most popular being XGBoost [31], CatBoost [39] and LightGBM [32]. In this study, we developed all models using the Python version of LightGBM 3.3.2.

### Loss functions

The default loss function for many gradient-based classifiers, including LightGBM, when dealing with imbalaced classification is the weighted cross-entropy (WCE) [22, 23], which measures how close the class probabilities predicted by the classifier match the true class labels. It is defined as follows:

1 $$\begin{array}{c}{L}_{CE}=-\sum_{n=1}^{m}{w}_{i}{y}_{n}\mathrm{log}(\widehat{{y}_{n}})+\left(1-{y}_{n}\right)\mathrm{log}\left(1-\widehat{{y}_{n}}\right)\end{array}$$

where $m$ is the total number of samples, ${y}_{n}$ are the target labels, $\widehat{{y}_{n}}$ are the predictions, ${w}_{i}$ is a tunable parameter to account for class imbalance. When handling imbalanced datasets, classifiers tend to disregard the first term, corresponding to mistakes on the minority class, and only focus on minimizing the second term, corresponding to mistakes on the majority class, leading to a suboptimal model [22, 23]. This can be tackled by setting ${w}_{i}$ equal to the ratio of inactive compounds versus active compounds.

#### Focal loss

Focal loss modifies the binary cross-entropy formulation by reducing the influence of well-classified samples on the overall loss [24, 38]. The formulation goes as follows:

2 $$\begin{array}{c}{L}_{F}=-\sum_{n=1}^{m}{y}_{n}{\left(1-\widehat{{y}_{n}}\right)}^{\gamma }\mathrm{log}\left(\widehat{{y}_{n}}\right)+\left(1-{y}_{n}\right){\widehat{{y}_{n}}}^{\gamma }\mathrm{log}\left(1-\widehat{{y}_{n}}\right)\end{array}$$

where $\gamma$ is a tunable parameter that affects the shape of the loss function. For high values of $\gamma$, the contribution of well classified samples to the overall loss approaches 0, allowing the gradient to focus more on the minority class. If $\gamma$ is set to 0, the focal loss coincides with the standard cross-entropy loss.

#### Logit-adjusted loss

Instead of modulating sample influence during the training process like weighted cross-entropy or Focal loss, Logit-adjusted loss scales the raw logits from the classifier according to the a priori probabilities of the classes [27], as shown in Formula 3

$$\begin{array}{c}{L}_{LA}=-\sum_{n=1}^{m}{y}_{n}\mathrm{log}\left(\sigma \left({p}_{n}+\tau *{\pi }_{m}\right)\right)+\left(1-{y}_{n}\right)\mathrm{log}\left(1-\sigma \left({p}_{n}+\tau *{\pi }_{M}\right)\right)\end{array}$$

where $\sigma$ is the sigmoid function, ${p}_{i}$ is the raw logit prediction, ${\pi }_{M}$ and ${\pi }_{m}$ are the prior probabilities for the majority and minority classes and $\tau$ is a smoothing factor that modulates the influence of the logit adjustments on the learning process. One key difference of Logit-adjusted loss compared to other approaches is that it guarantees Fisher consistency for the estimator by design, through a Bayes optimal solution for the balanced error. [27]

#### Label-distribution-aware margin loss

Similarly to Logit-adjusted loss, LDAM loss applies an offset to the raw logits from the model, but the optimal offsets are derived by minimizing a margin-based generalization bound [25]. One key limitation of margin-based approaches such as Support Vector Machines is that they rely on hinge loss [40], which is problematic to optimize for gradient-based methods because of its non-smoothness [25]. To tackle this issue, Cao et al. opted to use a cross-entropy inspired formulation, as shown in Formula 4:

4 $$\begin{array}{c}{L}_{LDAM}=-\sum_{n=1}^{m}{y}_{n}\mathrm{log}\left(\sigma \left({p}_{n}+\frac{C}{\sqrt[4]{{n}_{m}}}\right)\right)+\left(1-{y}_{n}\right)\mathrm{log}\left(1-\sigma \left({p}_{n}+\frac{C}{\sqrt[4]{{n}_{M}}}\right)\right)\end{array}$$

Where C is an hyperparameter to be tuned and ${n}_{m}$ and ${n}_{M}$ are the number of samples in the minority and majority class respectively.

#### Equalization loss

Another way to account for class imbalance is to operate at gradient level, for example by up-weighting gradients from minority samples and down-weighting the ones from majority samples according to the gradient ratio between classes. This approach has the theoretical advantage of weighting the minority class not only according to the class imbalance, but also according to the intrinsic difficulty of the classification problem, which might yield better weights compared to simple class counting statistics [26]. Another advantage is that this approach is function-agnostic, in the sense that it can be implemented to adjust any pre-existing loss function, i.e. cross-entropy.

To obtain the weighting coefficients for the gradients of the minority and majority classes, Equalization loss employs the following formula:

5 $$\begin{array}{c}{{w}_{m}}^{t}=1+\alpha \left(1-f\left({{g}_{r}}^{t}\right)\right)\end{array}$$

6 $$\begin{array}{c}{{w}_{M}}^{t}=f\left({{g}_{r}}^{t}\right)\end{array}$$

where ${{g}_{r}}^{t}$ is the ratio of accumulated gradients between the minority and majority classes at iteration $t$, $\alpha$ is a hyperparameter that allows to increase the weight for the minority class and $f$ is a mapping function:

7 $$\begin{array}{c}f\left(x\right)=\frac{1}{1+{e}^{-\gamma \left(x-\mu \right)}}\end{array}$$

With hyperparameters $\gamma$ and $\mu$.

To implement this approach, since Gradient Boosting is not trained with mini-batches, we considered the addition of one individual tree as one iteration, we clipped the gradients for numerical stability and we used binary cross-entropy as the underlying loss function.

### Datasets

To evaluate our proposed approach, we collected six datasets from publicly available and proprietary sources. From MoleculeNet [15] we selected Tox21, HIV and MUV, from MolData [20] we chose Phosphatase and NTPase and finally we added one high-troughput screening (HTS) dataset from Merck KGaA, resulting in approximately 2 million compounds and 42 tasks. This selection covers a broad imbalance range and dataset size, to ensure that our findings are not biased by specific dataset conditions.

To access the publicly available data, we downloaded the cleaned MoleculeNet datasets from Jiang et al. [13] and the MolData ones from Arshadi and coworkers. [20]

The datasets are summarized in Table 1, reporting the average number of compounds and imbalance ratios across tasks. The individual values pertaining each endpoint can be found in Additional file 1: Table S1. Since the HTS benchmark is a proprietary dataset from Merck KGaA, the exact number of compounds is confidential.

**Table 1:** Summary of the datasets employed in this study

Name	Source	Tasks	Compounds per task	Imbalance ratio
Tox21	MoleculeNet	12	6400	1:16
HIV	MoleculeNet	1	40748	1:27
MUV	MoleculeNet	17	14000	1:511
Phosphatase	MolData	5	330000	1:325
NTPase	MolData	6	330000	1:2963
HTS	Merck KGaA	1	> 330000	1:140

For a given dataset, the number of compounds per task and imbalance ratio are reported as averages across all tasks

### Metrics

A critical step of developing classifiers for imbalanced classification is the choice of metric to measure performance [41, 42]. For example, evaluating machine learning models according to accuracy when dealing with imbalanced data can lead to misleading conclusions, since it does not properly account for the performance on the minority class [5, 41, 42]. To allow for comparisons against the results previously reported in the literature for these benchmarks, we opted to evaluate all datasets using all metrics used by Arshadi et al. [20] and Jiang and coworkers [13], with the addition of balanced accuracy, F1 score and the Matthews correlation coefficient (MCC). Therefore, for each benchmark receiver operating characteristic area under curve (ROC-AUC), precision-recall area under curve (PR-AUC), accuracy, balanced accuracy, recall, precision, F1 score and MCC were measured. A more in-depth discussion on the choice of metrics and their definition can be found in: Sect. 1 of the. Given the number Additional file 1 information of classifiers and metrics involved in our study, for conciseness we show in the main text only the metrics reported by the authors of the respective benchmarks. The performance tables with all metrics employed in this study can be found in: Sect. 3, 4 and 5 of the Additional file 1 information

### Benchmarking procedure

After downloading the datasets from the respective repositories, all compounds were sanitized using RDKIT (version 2022.03.01) as described in the original papers and featurized using Extended-Connectivity Fingerprints (ECFP) with bit size 1024 and radius 2.

To develop the models, we followed two different benchmarking procedures depending on the dataset source. This way, the results obtained in this study are directly comparable to the performance of other classifiers reported in the respective papers. This enables us to put in perspective the improvements our approach provides over the default LightGBM implementation in a more conventional classifier comparison study.

For Tox21, HIV and MUV, we optimized each classifier in cross-validation using random splits, with a ratio of 80:10:10 for the training, validation and test set. Each model used early stopping on the loss of the validation set, while the test set was used to evaluate the performance of the model. To optimize the models we used Hyperopt (version 0.2.7) [43] for 20 iterations. Once the optimization was finished, we ran the model with optimal hyperparameters on 50 random splits, with a ratio of 80:10:10 for the training, validation and test set. Similar to the optimization phase, we used the validation set for early stopping and the test set for performance assessment. Regarding the choice of metrics, when comparing our approach to results from the literature we followed the guidelines from Wu et al. [15]: Tox21 and HIV were evaluated according to ROC-AUC, while MUV with PR-AUC.

For the Phosphatase and NTPase datasets, we employed the scaffold splits provided by Arshadi et al. [20] For each task, we optimized each model on the validation set and reported the performance on the test set. In all instances we used early stopping on the validation set to determine the optimal number of trees. All classifiers were optimized using Hyperopt [43] for 20 iterations and then evaluated 5 times using different random seeds. For comparisons with other machine learning algorithms, we reported the metrics employed by Arshadi et al. (accuracy, ROC-AUC, precision, recall) with the addition of the F1 score, to estimate the tradeoff between high precision and high recall.

For the Merck KGaA HTS dataset we employed the evaluation procedure for the MolData benchmarks. We created training, validation and testing sets using scaffold splitting with an 80:10:10 ratio. Then, we optimized all classifiers with Hyperopt for 20 iterations on the validation set using early stopping. Finally, we retrained each model with optimal parameters 5 times and measured all metrics on the test set.

To assess the efficacy of the custom loss functions, we use as baseline in all our benchmarks the performance of weighted cross-entropy and we evaluate whether the improvement is significant with 1-tailed Welch *t*-tests with Bonferroni correction. Furthermore, to contextualize the performance of LightGBM with custom loss functions, we compare the best performing model from our study to the models reported by Jiang et al. for MoleculeNet and by Arshadi et al. for MolData. All models from these papers employed weighted cross-entropy or class balancing schemes to model activity imbalance, depending on the underlying classification algorithm.

In the first study, four descriptor-based machine-learning methods and four graph-based neural networks were investigated. The descriptor-based models were Random Forest (RF), Support Vector Machine (SVM), XGBoost (XGB) and a neural network with dense layers (DNN), using a combination of 1D and 2D descriptors as well as two sets of fingerprints [13]. For the graph-based models, they considered a graph convolutional network (GCN), a graph attention network (GAT), a message-passing neural network (MPNN) and attentive fingerprints (AFP) [13]. For conciseness, for each MoleculeNet dataset we report the performance of the best descriptor-based model and graph-based model and we compare them to the best-performing LightGBM model using 2-tailed Welch *t*-tests with Bonferroni correction.

In the second study, the authors developed a multitask DNN on ECFP fingerprints with bit size 1024 and radius 2 and a multitask GCN. For these baselines, we omit statistical tests since the authors did not report standard deviations for their results.

The benchmarking details for all datasets are summarized in Table 2.

**Table 2:** Summary of the benchmarking procedure for each dataset employed in this study

Name	Split	Replicates	Metrics for external comparison	External baselines
HIV	Random	50	ROC-AUC	RF, SVM, XGB, DNN, GCN, GAT, MPNN, AFP
Tox21	Random	50	ROC-AUC	RF, SVM, XGB, DNN, GCN, GAT, MPNN, AFP
MUV	Random	50	PR-AUC	RF, SVM, XGB, DNN, GCN, GAT, MPNN, AFP
Phosphatase	Scaffold	5	Accuracy, precision, recall, F1 score, ROC-AUC	DNN, GCN
NTPase	Scaffold	5	Accuracy, precision, recall, F1 score, ROC-AUC	DNN, GCN
HTS	Scaffold	5	Not applicable	Not applicable

## Results

### Moleculenet benchmarks

**Fig. 1:**
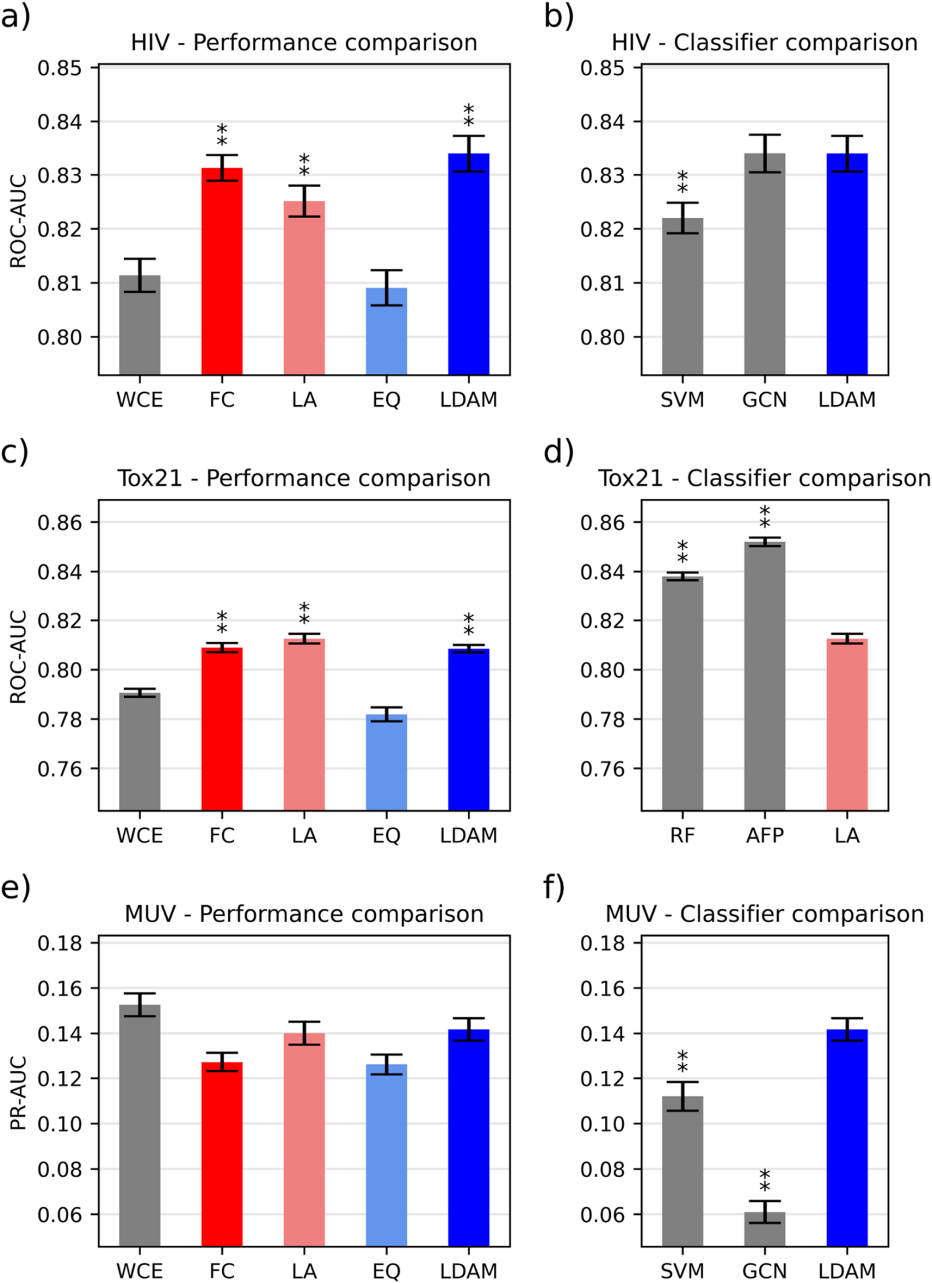
Summary of the benchmarking results for the MoleculeNet datasets. Error bars represent the standard error of the mean (N = 50), while the asterisks denote whether the difference is significant (one indicates α < 0.05, two α < 0.01). The statistical tests with Bonferroni correction are carried out with respect to WCE or to the best performing loss function. We define the differences between loss functions within LightGBM as performance comparisons, while classifier comparisons refer to the benchmarking of the best loss function against the classifiers from Jiang et al. **a** Loss function comparison on the HIV dataset. **b** Comparison between the best loss function and the best models from Jiang et al. on the HIV dataset **c** Loss function comparison on the Tox21 dataset. **d** Comparison between the best loss function and the best models from Jiang et al. on the Tox21 dataset. **e** Loss function comparison on the MUV dataset. **f** Comparison between the best loss function and the best models from Jiang et al. on the MUV dataset

The results for the datasets from MoleculeNet are summarized in Table 3 and Fig. 1, while the *p*-values for the statistical tests are outlined in Additional file 1: Tables S8, S9, S10 and S14. The performance across all metrics for these datasets is shown in Additional file 1: Tables S2, S3 and S4.

**Table 3:** Summary of the results for the datasets belonging to the MoleculeNet repository

Name	Metric	WCE	FC	LA	EQ	LDAM	Best descriptor-based	Best graph-based
HIV	ROC-AUC	0.811 ± 0.02	0.831 ± 0.01	0.823 ± 0.03	0.809 ± 0.02	**0.833 ± 0.02**	0.822 ± 0.02	**0.833 ± 0.02**
Tox21	ROC-AUC	0.790 ± 0.01	0.808 ± 0.01	0.812 ± 0.01	0.781 ± 0.02	0.808 ± 0.01	0.838 ± 0.01	**0.852 ± 0.01**
MUV	PR-AUC	**0.152 ± 0.03**	0.127 ± 0.02	0.140 ± 0.03	0.126 ± 0.03	0.141 ± 0.03	0.112 ± 0.04	0.061 ± 0.03

The best values for each metric in each dataset are highlighted in bold

Focal loss, Logit-adjusted loss and LDAM loss significantly outperform the weighted cross-entropy baseline for the HIV dataset. The best performing loss function is LDAM loss (0.833 ROC AUC), closely followed by Focal loss. Equalization loss achieves the lowest ROC-AUC out of all custom loss functions. Considering all metrics, Focal loss achieves the best performance in terms of PR-AUC, accuracy, F1 score and MCC and Equalization loss achieves the best precision value. With the exception of the F1 score, all differences are statistically significant. In terms of recall and balanced accuracy however, WCE outperforms all alternatives. Compared to the best descriptor-based model (SVM) and graph-based model (GCN) from Jiang et al., the LightGBM model with LDAM loss significantly outperforms the former and matches the ROC-AUC from the latter. The improvement on this dataset is especially significant, given that the weighted cross-entropy baseline is outperformed by both alternatives from Jiang et al.

For Tox21, similarly to the previous dataset, all custom losses with the exception of Equalization loss significantly outperform the weighted cross-entropy baseline in terms of ROC-AUC. Logit-adjusted loss achieves the best ROC-AUC with 0.812, narrowly outperforming LDAM loss and Focal loss. In terms of global performance however, LDAM loss has the most success, outperforming all alternatives on four metrics (PR-AUC, accuracy, precision, MCC), but except for precision and accuracy the differences are not statistically significant compared to the baseline. WCE achieves the best performance in terms of balanced accuracy, recall and F1 score. When comparing to the best models from Jiang et al., both options (RF and AFP) significantly outperform the Gradient Boosting classifier with Logit-adjusted loss, possibly pointing to the fact that LightGBM might not be a good option for this dataset. Unlike XGBoost, LightGBM employs a leaf-wise tree splitting procedure, which is known to potentially lead to more complex structures that might overfit on small datasets [31, 32]. Among the datasets tested, Tox21 has the least compounds per task, which might explain why LightGBM performs comparatively poorly.

Regarding MUV, none of the custom losses are able to outperform the weighted cross-entropy baseline in any metric except accuracy. This is especially surprising considering that MUV is the most imbalanced dataset considered in this study, where one would expect to observe the greatest improvement over the baseline. This could be explained by the fact that the custom loss functions must optimize additional hyperparameters related to the loss, which have a strong impact on the performance of the classifier [27]. Since all classifiers generally achieve low PR-AUC values for this dataset, tuning these additional parameters could lead to a very noisy optimization process leading to an inferior optimum for a given number of iterations. Increasing the number of optimization evaluations could mitigate this issue.

Among the custom loss functions, LDAM loss performs the best with a PR-AUC value of 0.141, closely followed by Logit-adjusted loss. Interestingly, all LightGBM models are able to outperform all models from Jiang et al. Indeed, for this dataset LightGBM achieves more than double the performance reported for XGBoost in their paper. This again could be related to the differences in the tree-splitting procedure between the two implementations. Finally, the dataset also highlights the issues of data-driven representations when dealing with extreme imbalance, since in this benchmark all graph-based approaches achieve substantially lower performance than descriptor-based classifiers.

### Moldata benchmarks

The custom loss functions were next evaluated using the MolData datasets.

**Fig. 2:**
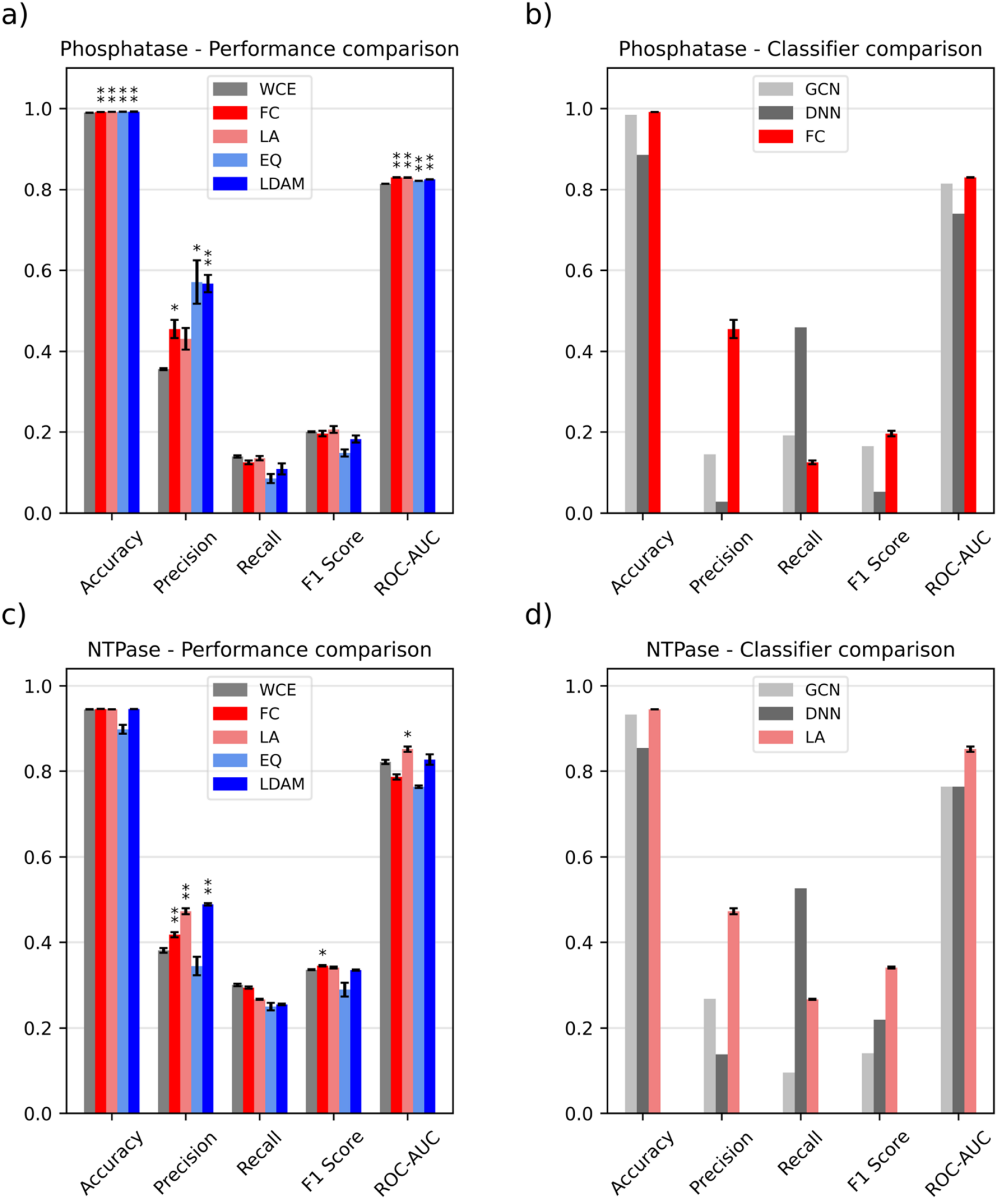
Summary of the benchmarking results for the MolData datasets. Error bars represent the standard error of the mean (N = 5), while the asterisks denote whether the difference is significant (one indicates α < 0.05, two α < 0.01). The statistical tests with Bonferroni correction are carried out with respect to WCE. We define the differences between loss functions within LightGBM as performance comparisons, while classifier comparisons refer to the benchmarking of the best loss function against the classifiers from Arshadi et al. **a** Loss function comparison on the Phosphatase dataset. **b** Comparison between the best loss function and the best models from Arshadi et al. on the Phosphatase dataset **c** Loss function comparison on the NTPase dataset. **d** Comparison between the best loss function and the best models from Arshadi et al. on the NTPase dataset

All custom loss functions significantly outperform the weighted cross-entropy baseline for the Phosphatase dataset in terms of accuracy, precision (except Logit-adjusted loss) and ROC-AUC (Table 4, Additional file 1: Table S5 and Fig. 2, *p*-values for the statistical tests outlined in Additional file 1: Table S11). The only metrics where the baseline still outperforms the alternatives are recall and balanced accuracy. The F1 score for Logit-adjusted loss is higher, indicating that the trade-off between precision and recall is generally favorable, however the difference is not statistically significant. In terms of MCC and PR-AUC, LA loss achieves the best performance, significantly outperforming the baseline on both metrics. Compared to the multitask networks from Arshadi and coworkers, Focal loss outperforms them in all metrics except recall. The improvement is especially noticeable in terms of precision, achieving more than double the value reported for the GCN model.

**Table 4:** Summary of the benchmarking results for the datasets in the MolData repository

Name	Metric	WCE	FC	LA	EQ	LDAM	DNN—Arshadi	GCN –Arshadi
Phosphatase	Accuracy	0.989 ± 0.0005	**0.992 ± 4E-4**	**0.992 ± 3E-4**	**0.992 ± 7E-4**	**0.992 ± 2E-4**	0.885	0.984
	Precision	0.356 ± 0.01	0.455 ± 0.05	0.431 ± 0.06	**0.571 ± 0.01**	0.567 ± 0.05	0.027	0.144
	Recall	0.139 ± 0.006	0.125 ± 0.01	0.135 ± 0.01	0.085 ± 0.02	0.109 ± 0.03	**0.459**	0.191
	F1 score	0.200 ± 0.003	0.196 ± 0.01	**0.206 ± 0.01**	0.148 ± 0.01	0.182 ± 0.02	0.052	0.164
	ROC-AUC	0.814 ± 0.0005	**0.830 ± 0.001**	**0.830 ± 0.01**	0.821 ± 0.0003	0.825 ± 0.0008	0.739	0.815
NTPase	Accuracy	0.945 ± 0.001	0.945 ± 0.004	0.945 ± 0.0004	0.899 ± 0.02	**0.946 ± 0.005**	0.854	0.933
	Precision	0.381 ± 0.01	0.417 ± 0.01	0.472 ± 0.01	0.344 ± 0.04	**0.488 ± 0.006**	0.138	0.267
	Recall	0.300 ± 0.007	0.294 ± 0.005	0.267 ± 0.003	0.250 ± 0.02	0.255 ± 0.005	**0.526**	0.095
	F1 score	0.336 ± 0.003	**0.345 ± 0.004**	0.341 ± 0.005	0.289 ± 0.03	0.335 ± 0.003	0.219	0.141
	ROC-AUC	0.821 ± 0.01	0.787 ± 0.01	**0.852 ± 0.01**	0.764 ± 0.007	0.827 ± 0.02	0.763	0.763

The best values for each metric in each dataset are highlighted in bold

For the NTPase benchmark, Logit-adjusted loss stands out as the best option, significantly outperforming the baseline in terms of precision, ROC-AUC and MCC (Table 4, Additional file 1: Table S6 and Fig. 2, p-values in Additional file 1: Table S12). LDAM loss and Focal loss also improve over the baseline, but the trend is not as consistent as for Logit-adjusted loss across all metrics. When comparing it to the baselines from Arshadi and coworkers, similarly to the results for the Phosphatase dataset, Logit-adjusted loss outperforms both multitask networks in all metrics except recall. The improvement is especially noticeable for ROC-AUC, going from 0.76 to 0.85.

### Proprietary dataset benchmark

**Fig. 3:**
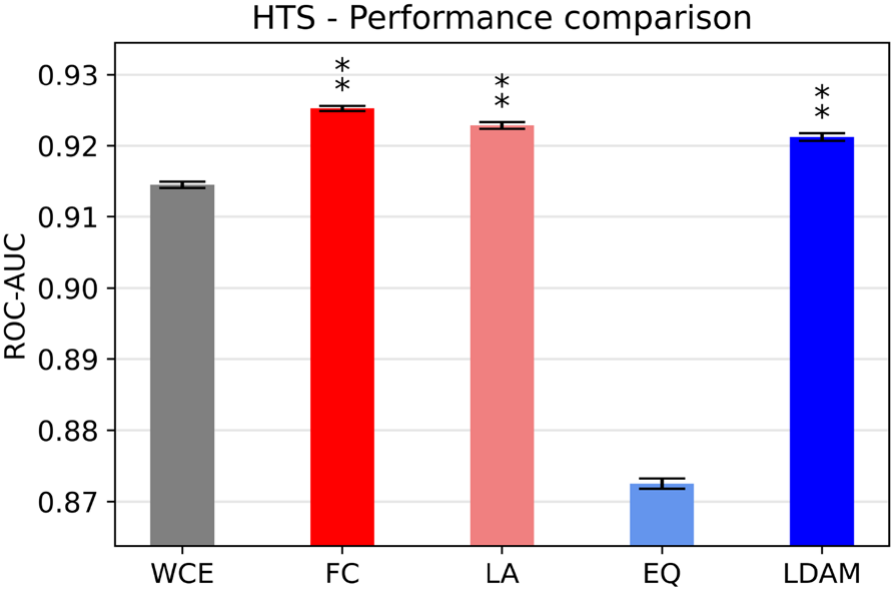
Benchmarking results for the proprietary HTS dataset. Error bars represent the standard error of the mean (N = 5), while the asterisks denote whether the difference is significant (one indicates α < 0.05, two α < 0.01). The statistical tests are carried out with respect to WCE

All loss functions, except Equalization loss, achieve excellent performance on the real-world industrial dataset, with ROC-AUC values above 0.9 (Fig. 3 and Additional file 1: Table S14, p-values for the statistical tests can be found in Additional file 1: Table S15). Focal loss, LDAM loss and Logit-adjusted loss significantly outperform the weighted cross-entropy baseline, consistently with the trends observed in the academic datasets. However, the relative increases between the baseline and the custom loss functions are minimal in terms of magnitude. This is likely because these classifiers already achieve near perfect performance, making it difficult to achieve substantial improvements. Considering the other metrics, Focal loss achieves the best performance on all metrics except balanced accuracy and recall, significantly outperforming the baseline in PR-AUC, precision, F1 score, MCC and accuracy. Logit-adjusted loss performs similarly to Focal loss, matching its performance in terms of MCC and PR-AUC while obtaining higher balanced accuracy.

### Influence on convergence speed

**Fig. 4:**
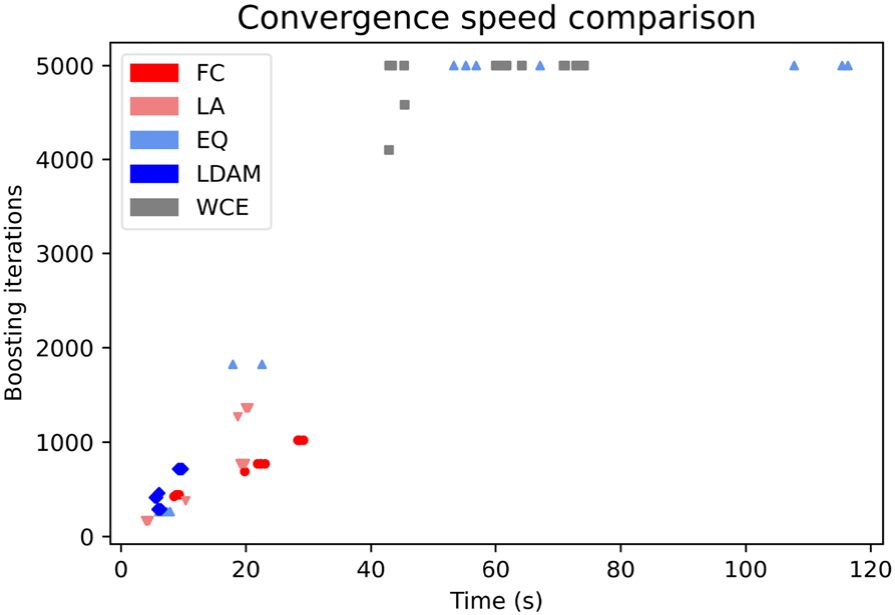
Convergence speed comparison between weighted cross-entropy and the custom loss functions. Each dot represents a fit iteration in terms of boosting iterations required to trigger early stopping and computational time

To assess whether changing the loss function affects the number of boosting iterations required for convergence, we analyzed the number of trees and time required to fit the HIV dataset for each loss function. To do so, we optimized the hyperparameters of each classifier and measured the training time and number of trees on five 80:20 training-validation splits, using the external set for early stopping. The whole procedure was repeated three times, to ensure that the findings are independent of specific optima obtained during the optimization phase, for a total of 15 measurements per loss function. The results are summarized in Fig. 4, Additional file 1: Table S16 and Additional file 1: Table S17. Interestingly, the weighted cross-entropy baseline is the most computationally expensive option on average, requiring on average around 4900 boosting iterations and 59 s to fit the dataset. LDAM loss is the fastest loss function on average (7 s), closely followed by Logit-adjusted loss (13 s) and Focal loss (19 s). Equalization loss has the widest spread in terms of boosting iterations and training time, likely arising from training instability for this loss function.

## Discussion

Remarkably, on five out of six datasets investigated, at least one custom loss function outperformed the weighted cross-entropy baseline. These findings display that our approach is robust to a wide variety of endpoints, dataset sizes and imbalance rates, including real world data. On average, the Equalization loss performed the worst, while Logit-adjusted loss achieved consistently strong performance across all datasets, followed by LDAM loss and Focal loss.

One possible explanation for the lower effectiveness of Equalization loss might be that approximating one mini-batch with the fitting of one boosted tree is not appropriate, thus rendering the accumulated gradient ratios unreliable. This is further confirmed by the high instability of the gradients we observed while implementing this loss for Gradient Boosting, which we attempted to correct using gradient clipping. Moreover, it is interesting that this custom loss function, which is the most similar to weighted cross-entropy since it relies on dynamically weighting the two class contributions, is also the one achieving the poorest performance. This further highlights the need for alternative approaches such as applying a class-specific offset to the raw logits (LDAM loss and Logit-adjusted loss), or dampening the influence of well-classified samples (Focal loss).

When analyzing our results across all metrics, one relevant finding is that using custom loss functions leads to an overall increase in precision at the expense of recall when comparing to the weighted cross-entropy baseline. Depending on the context and purpose for which these datasets are modeled, the increase in precision might be extremely beneficial, i.e. in settings where experimental testing is expensive so it is paramount to reduce the number of false positives. Another interesting trend is the systematic increase in accuracy compared to the baseline, however this is not significant considering the inadequacy of this metric for imbalanced classification. In terms of global performance however, our proposed modifications still lead to better models overall, as indicated by generally higher MCC, ROC-AUC, PR-AUC and F1 scores across five out of six datasets. Furthermore, the increase in performance in terms of MCC is especially significant, given that this metric is known to perform extremely well in ranking classifiers when dealing with class imbalance [41]. It should be noted however that if the target metric is balanced accuracy, the baseline would be a more indicated choice of loss function since it consistently outperforms all alternatives.

Regarding the comparison with the external baselines from Arshadi et al. and Jiang et al., implementing the custom loss functions discussed in this study allows LightGBM to match or outperform the best models from those studies in four out of five datasets. This result is noteworthy considering the wide variety and complexity of the approaches employed by Jiang et al. and the fact that Gradient Boosting does not benefit from multitask learning, unlike the approaches from Arshadi et al. These findings highlight the importance of properly addressing imbalance with bespoke approaches rather than relying on simpler loss weighting schemes.

Regarding the convergence time, all losses required less iterations and training time than the weighted cross-entropy baseline, speeding up the computation by a factor of 8 for LDAM loss, 4 for Logit-adjusted loss, 3 for Focal loss and 1.2 for Equalization loss. One possible explanation for this could be that the modifications of cross-entropy investigated in this study provide more informative gradients, leading to faster convergence [44, 45]. This phenomenon could be caused by the inclusion of prior class probabilities in the loss formulation (Logit-adjusted and LDAM losses), or by forcing the total loss to be more dependent on hard to classify examples (Focal loss).

In summary, considering both the performance improvement and the influence on convergence time, Logit-adjusted and LDAM loss are the best options for tuning Gradient Boosting for imbalanced bioassay modelling. Interestingly, both approaches rely on logit shifting, which seems to indicate that this strategy is preferable than weighting approaches like Equalization loss or Focal loss, in agreement with the findings from Menon and coworkers [27]. Furthermore, both options, given sufficient hyperparameter optimization, can converge back to the original cross-entropy formulation, meaning that they are a suitable option even on datasets where the baseline might achieve better performance.

Finally, LightGBM with these modifications is a strong, efficient and interpretable baseline for future works on ligand-based virtual screening. This will provide an out-of-the-box solution for quickly modelling large bioassay data and will serve as a meaningful benchmark for more complex algorithms on imbalanced datasets.

## Conclusion

In this study, we investigated the effectiveness of custom loss functions applied to Gradient Boosting for modelling extremely imbalanced bioassay data. To answer this question, we evaluated our approach against weighted cross-entropy, the current de-facto standard for imbalanced data classification, and a variety of classifiers from previous studies involving approximately 2 million compounds and 42 tasks from public and proprietary sources.

Our results show that all bespoke loss functions achieve statistically significant improvement over weighted cross-entropy across 5 out of 6 benchmarks, the most promising being Logit-adjusted loss and LDAM loss. Furthermore, thanks to these modifications, Gradient Boosting is able to match or outperform the best classifiers of other benchmarks for four out of five datasets. Additionally, the use of custom loss reduces the training time and computational cost for gradient boosting, as highlighted in our convergence iteration comparison.

The significance of these results is three-fold. First, they show the importance of appropriately tackling class imbalance with custom loss functions, an approach that has not been thoroughly investigated in the context of drug discovery until now. These modifications are particularly promising considering their widespread success in computer vision and could substitute or complement resampling-based approaches, which are already well established for bioassay modelling [5, 29, 30]. Second, they highlight the efficacy of Gradient Boosting coupled with proper loss functions for modelling extremely imbalanced bioassay data. This is relevant because Gradient Boosting has a unique set of advantages over other classifiers such as excellent scalability to large datasets [31, 32, 39], straightforward interpretability [17] and ease of optimization [19]. Third, our analysis shows that logit-shifting modifications of the cross-entropy loss are generally more performant than weighting-based approaches for gradient boosting. This provides a solid foundation for developing novel loss functions and simplifies the choice of loss function when modelling imbalanced data.

Finally, our implementation, available at https://github.com/dahvida/gradient_boosting_CLF, is designed to handle any function definition with minimal external package dependencies to streamline the implementation of alternative loss functions for Gradient Boosting. We hope this will accelerate further research on newer loss functions for class imbalance, i.e. combo losses [46], as well as for regular classification, for example 0–1 losses with Langevin gradient descent [47].

## Supplementary Information

**Additional file 1: Table S1.** Description of the number of compounds and imbalance ratio, defined as the number of inactive compounds divided by the number of active ones, for each endpoint in each dataset. **Table S2.** Summary of the benchmarking results for the HIV dataset. The best values for each metric in each dataset are highlighted in bold. **Table S3.** Summary of the benchmarking results for the Tox21 dataset. The best values for each metric in each dataset are highlighted in bold. **Table S4.** Summary of the benchmarking results for the MUV dataset. The best values for each metric in each dataset are highlighted in bold. **Table S5.** Summary of the benchmarking results for the Phosphatase dataset. The best values for each metric in each dataset are highlighted in bold. **Table S6.** Summary of the benchmarking results for the NTPase dataset. The best values for each metric in each dataset are highlighted in bold. **Table S7.** Significance levels for the Welch tests after Bonferroni correction for each dataset. **Table S8.** P-values of the Welch tests (N = 50) for the HIV dataset against WCE. **Table S9.** P-values of the Welch tests (N = 50) for the Tox21 dataset against WCE. **Table S10.** P-values of the Welch tests (N = 50) for the MUV dataset against WCE. **Table S11.** P-values of the Welch tests (N = 5) for the Phosphatase dataset against WCE. **Table S12.** P-values of the Welch tests (N = 5) for the NTPase dataset against WCE. **Table S13.** P-values of the Welch tests (N = 50) for the datasets from the MoleculeNet repository against the models from Arshadi et al. **Table S14.** Summary of the benchmarking results for the HTS dataset. The best values for each metric in each dataset are highlighted in bold. **Table S15.** P-values of the Welch tests (N = 5) for the HTS dataset against WCE. **Table S16.** Boosting iterations for each loss function with optimal hyperparameters for the HIV dataset. **Table S17.** Boosting iterations for each loss function with optimal hyperparameters for the HIV dataset.

## Data Availability

The full data and the code required to reproduce the results described in this study are available at the following github repository: https://github.com/dahvida/gradient_boosting_CLF.

## Abbreviations

ML: Machine learning
DL: Deep learning
AE: Autoencoder
GNN: Graph neural network
FC: Focal loss
LA: Logit-adjusted loss
EQ: Equalization loss
LDAM: Label-distribution aware margin loss
WCE: Weighted cross-entropy
ECFP: Extended connectivity fingerprint
RF: Random forest
SVM: Support Vector machine
XGB: XGBoost
DNN: Dense neural network
GCN: Graph convolutional neural network
GAT: Graph attention neural network
MPNN: Message-passing neural network
AFP: Attentive fingerprint
ROC-AUC: Receiver operating characteristic area under curve
PR-AUC: Precision–recall area under curve
