# Supplementary material for: Tuning gradient boosting for imbalanced bioassay modelling with custom loss functions
Source: J Cheminform. 2022 Nov 10;14:80. doi: 10.1186/s13321-022-00657-w (PMC9650867; doi:10.1186/s13321-022-00657-w)
Supplement: Supplementary file 1 — Additional file 1: Table S1. Description of the number of compounds and imbalance ratio, defined as the number of inactive compounds divided by the number of active ones, for each endpoint in each dataset. Table S2. Summary of the benchmarking results for the HIV dataset. The best values for each metric in each dataset are highlighted in bold. Table S3. Summary of the benchmarking results for the Tox21 dataset. The best values for each metric in each dataset are highlighted in bold. Table S4. Summary of the benchmarking results for the MUV dataset. The best values for each metric in each dataset are highlighted in bold. Table S5. Summary of the benchmarking results for the Phosphatase dataset. The best values for each metric in each dataset are highlighted in bold. Table S6. Summary of the benchmarking results for the NTPase dataset. The best values for each metric in each dataset are highlighted in bold. Table S7. Significance levels for the Welch tests after Bonferroni correction for each dataset. Table S8. P-values of the Welch tests (N = 50) for the HIV dataset against WCE. Table S9. P-values of the Welch tests (N = 50) for the Tox21 dataset against WCE. Table S10. P-values of the Welch tests (N = 50) for the MUV dataset against WCE. Table S11. P-values of the Welch tests (N = 5) for the Phosphatase dataset against WCE. Table S12. P-values of the Welch tests (N = 5) for the NTPase dataset against WCE. Table S13. P-values of the Welch tests (N = 50) for the datasets from the MoleculeNet repository against the models from Arshadi et al. Table S14. Summary of the benchmarking results for the HTS dataset. The best values for each metric in each dataset are highlighted in bold. Table S15. P-values of the Welch tests (N = 5) for the HTS dataset against WCE. Table S16. Boosting iterations for each loss function with optimal hyperparameters for the HIV dataset. Table S17. Boosting iterations for each loss function with optimal hyperparameters for the HIV dataset. [file 13321_2022_657_MOESM1_ESM.docx]

**Additional file information**

Tuning gradient boosting for imbalanced bioassay modelling with custom loss functions

Davide Boldini,^†^ Lukas Friedrich,^‡^ Daniel Kuhn^‡^ and Stephan A. Sieber*^,†^

†Center for functional Protein Assemblies, Technical University of Munich (TUM), Ernst-Otto-Fischer-Straße 8, Garching, D-85784, Germany

‡Merck Healthcare KGaA, Frankfurter Straße 250, Darmstadt, D-64293, Germany

1. **Evaluation metrics**

A key aspect of imbalanced classification is the choice of metrics to evaluate the performance of the machine learning model.^1^ Because of the skewed class distribution, popular metrics like accuracy tend to yield over-optimistic results and ignore the performance on the minority class.^2^

Furthermore, depending on the intended use of the machine learning model, different aspects of its predictive capability might be prioritized.^1^ For example, when dealing with toxicity prediction, false positives have lower priority than false negatives because of the health implications when not correctly identifying a toxic compound. On the other hand, during virtual screening campaigns, having a high false positive rate can be problematic if the assay procedure to experimentally verify the predictions is lengthy or expensive. Therefore, depending on the application of interest, different metrics might be more appropriate to characterize the classifier’s performance.

As such, to accurately evaluate our proposed approach under all possible use cases for imbalanced classification we chose to employ a panel of 8 classification metrics on all benchmarks. Our selection encompasses all metrics indicated by the benchmarks’ original authors to be able to compare our results with other studies.^3,4^ We also add additional figures of merit to further characterize the performance of the classifiers, in accordance with recent publications on the topic.^1,2^

We provide the mathematical formulation and a short introduction to each metric below.

**Abbreviations:**

FP = false positive

TP = true positive

FN = false negative

TN = true negative

TPR = true positive rate

TNR = true negative rate

- **Accuracy**

$$\begin{aligned} Accuracy= \frac{TP+TN}{TP+TN+FP+FN}\#1.1 \end{aligned}$$

Accuracy is defined as the ratio between correct predictions and all predictions. If the dataset is imbalanced (i.e. the number of active compounds is much smaller than the number of inactives), the metric becomes almost exclusively dependent on TN, not capturing effectively the performance of the classifier on the minority class. We decided to keep it to be consistent with the metric choice of the authors of the MolData benchmarking suite.^4^

- **Recall**

$$\begin{aligned} Recall=\frac{TP}{TP+FN}\#1.2 \end{aligned}$$

Recall is defined as the true positive ratio, or the number of positive samples correctly identified out of all positive samples in the dataset. Since this metric is class-specific, it can be a useful indicator to estimate the detection rate of the minority class.

- **Precision**

$$\begin{aligned} Precision= \frac{TP}{TP+FP}\#1.3 \end{aligned}$$

Precision is defined as the fraction of true positive samples out of all positive predictions. Like recall, this metric is class-specific and it can be used to evaluate the likelihood of incurring in false positives on the minority class.

- **F1 Score**

$$\begin{aligned} F1 Score=2*\frac{Precision*Recall}{Precision+Recall}\#1.4 \end{aligned}$$

The F1 Score is defined as the harmonic mean between precision and recall. Maximizing precision and recall simultaneously tends to be quite challenging when modelling imbalanced datasets, as such most classifiers exhibit either high precision or high recall. The F1 Score provides an unbiased statistic of this trade-off, since it includes both metrics but punishes extremely low values in either term.^1^

- **Balanced accuracy**

$$\begin{aligned} Balanced accuracy= \frac{1}{2}*\left( \frac{TP}{TP+FN}+\frac{TN}{TN+FP} \right)=\frac{1}{2}*\left( TPR+TNR \right)\#1.5 \end{aligned}$$

The balanced accuracy can be defined as the mean recall of the minority and majority classes, or as the mean between the true positive rate and true negative rate. Unlike accuracy, this metric is not sensitive to class imbalance and given that it considers both classes, it can be used to globally assess the performance of a classifier for imbalanced classification.

- **Matthews correlation coefficient (MCC)**

$$\begin{aligned} MCC=\frac{TP*TN-FP*FN}{\sqrt{\left( TP+FP \right)\left( TP+FN \right)\left( TN+FP \right)\left( TN+FN \right)}}\#1.6 \end{aligned}$$

The Matthews correlation coefficient is defined between -1 and 1, with -1 indicating perfect inverse correlation, 0 no correlation and 1 perfect correlation. It is considered to be one of the best indices for imbalanced classification, improving over the F1 score by taking into account the number of true negatives into its formulation.^2^

- **Receiver operating characteristic area under curve (ROC-AUC)**

Unlike previous metrics, ROC-AUC is computed on raw probability scores instead of the labels predicted by the classifier. The construction of the curve goes as follow. First, a set of possible probability thresholds is defined to convert the raw probability scores into predicted labels. Then, for each threshold the false positive rate (defined as 1-TNR) and the true positive rate are plotted on the x and y axis respectively. Finally, the area under the curve is calculated to summarize the performance of the classifier at every possible threshold. This value then is defined between 0 and 1: 0 indicates perfect inverse correlation, 0.5 no correlation and 1 perfect correlation. Because of the values it adopts on its axes, ROC-AUC is closely related to balanced accuracy.

- **Precision – recall area under curve (PR-AUC)**

PR-AUC is computed in a similar manner to ROC-AUC, the only difference being the substitution of the true positive rate with precision. While being also defined between 0 and 1, unlike ROC-AUC a score of 0 indicates no correlation. While ROC-AUC is known to be over-optimistic when dealing with extreme imbalance levels, PR-AUC is a more robust indicator for such scenarios.^5^

1. **Full dataset information**

**Additional file 1: Table S1** – Description of the number of compounds and imbalance ratio, defined as the number of inactive compounds divided by the number of active ones, for each endpoint in each dataset.

| **Dataset** | **Task number** | **α = 0.05** | **α = 0.01** |
| --- | --- | --- | --- |
| HIV | 0 | 40748 | 27.7 |
| Tox21 | 0 | 7265 | 22.5 |
| Tox21 | 1 | 6758 | 27.5 |
| Tox21 | 2 | 6549 | 7.5 |
| Tox21 | 3 | 5821 | 18.4 |
| Tox21 | 4 | 6193 | 6.8 |
| Tox21 | 5 | 6955 | 18.9 |
| Tox21 | 6 | 6450 | 33.7 |
| Tox21 | 7 | 5832 | 5.2 |
| Tox21 | 8 | 7072 | 25.8 |
| Tox21 | 9 | 6467 | 16.4 |
| Tox21 | 10 | 5810 | 5.3 |
| Tox21 | 11 | 6774 | 15.0 |
| MUV | 0 | 14844 | 548.8 |
| MUV | 1 | 14737 | 507.2 |
| MUV | 2 | 14734 | 490.1 |
| MUV | 3 | 14633 | 486.8 |
| MUV | 4 | 14903 | 512.9 |
| MUV | 5 | 14606 | 502.7 |
| MUV | 6 | 14647 | 487.2 |
| MUV | 7 | 14415 | 513.8 |
| MUV | 8 | 14841 | 510.8 |
| MUV | 9 | 14691 | 523.7 |
| MUV | 10 | 14696 | 505.8 |
| MUV | 11 | 14646 | 504.0 |
| MUV | 12 | 14676 | 488.2 |
| MUV | 13 | 14714 | 489.5 |
| MUV | 14 | 14658 | 504.4 |
| MUV | 15 | 14775 | 508.5 |
| MUV | 16 | 14751 | 613.6 |
| Phos | 0 | 260322 | 576.2 |
| Phos | 1 | 260322 | 121.6 |
| Phos | 2 | 298215 | 526.8 |
| Phos | 3 | 295459 | 416.3 |
| Phos | 4 | 280994 | 123.8 |
| NTP | 0 | 269407 | 360.6 |
| NTP | 1 | 257568 | 5.5 |
| NTP | 2 | 251895 | 3.9 |
| NTP | 3 | 283069 | 384.1 |
| NTP | 4 | 276522 | 16265.0 |
| NTP | 5 | 301932 | 298.8 |

1. **Performance tables**

**Additional file 1: Table S2** - Summary of the benchmarking results for the HIV dataset. The best values for each metric in each dataset are highlighted in bold.

| **Metric** | **FC** | **LA** | **LDAM** | **EQ** | **WCE** |
| --- | --- | --- | --- | --- | --- |
| ROC-AUC | 0.831 ± 0.01 | 0.823 ± 0.03 | **0.833 ± 0.02** | 0.809 ± 0.02 | 0.811 ± 0.02 |
| PR-AUC | **0.49 ± 0.041** | 0.459 ± 0.041 | 0.482 ± 0.046 | 0.351 ± 0.055 | 0.467 ± 0.04 |
| Accuracy | **0.972 ± 0.002** | 0.971 ± 0.003 | 0.971 ± 0.003 | 0.969 ± 0.002 | 0.969 ± 0.003 |
| Balanced accuracy | 0.694 ± 0.029 | 0.696 ± 0.031 | 0.701 ± 0.031 | 0.605 ± 0.04 | **0.706 ± 0.027** |
| Precision | 0.669 ± 0.065 | 0.615 ± 0.065 | 0.636 ± 0.084 | **0.685 ± 0.095** | 0.585 ± 0.066 |
| Recall | 0.395 ± 0.059 | 0.401 ± 0.064 | 0.411 ± 0.066 | 0.214 ± 0.082 | **0.424 ± 0.057** |
| F1 score | **0.493 ± 0.047** | 0.48 ± 0.043 | 0.492 ± 0.041 | 0.314 ± 0.092 | 0.487 ± 0.038 |
| MCC | **0.499 ± 0.043** | 0.479 ± 0.038 | 0.493 ± 0.038 | 0.361 ± 0.07 | 0.48 ± 0.037 |

**Additional file 1: Table S3** - Summary of the benchmarking results for the Tox21 dataset. The best values for each metric in each dataset are highlighted in bold.

| **Metric** | **FC** | **LA** | **LDAM** | **EQ** | **WCE** |
| --- | --- | --- | --- | --- | --- |
| ROC-AUC | 0.808 ± 0.01 | **0.812 ± 0.01** | 0.808 ± 0.01 | 0.781 ± 0.02 | 0.790 ± 0.01 |
| PR-AUC | 0.448 ± 0.019 | 0.445 ± 0.022 | **0.462 ± 0.026** | 0.415 ± 0.022 | 0.452 ± 0.024 |
| Accuracy | 0.929 ± 0.004 | 0.928 ± 0.004 | **0.931 ± 0.004** | 0.921 ± 0.01 | 0.923 ± 0.006 |
| Balanced accuracy | 0.663 ± 0.009 | 0.66 ± 0.014 | 0.661 ± 0.016 | 0.602 ± 0.013 | **0.68 ± 0.014** |
| Precision | 0.603 ± 0.043 | 0.605 ± 0.047 | **0.642 ± 0.06** | 0.463 ± 0.063 | 0.559 ± 0.044 |
| Recall | 0.353 ± 0.02 | 0.348 ± 0.031 | 0.348 ± 0.035 | 0.232 ± 0.034 | **0.395 ± 0.032** |
| F1 score | 0.414 ± 0.021 | 0.413 ± 0.028 | 0.417 ± 0.035 | 0.271 ± 0.029 | **0.431 ± 0.026** |
| MCC | 0.409 ± 0.022 | 0.407 ± 0.028 | **0.419 ± 0.035** | 0.271 ± 0.028 | 0.414 ± 0.026 |

**Additional file 1: Table S4** - Summary of the benchmarking results for the MUV dataset. The best values for each metric in each dataset are highlighted in bold.

| **Metric** | **FC** | **LA** | **LDAM** | **EQ** | **WCE** |
| --- | --- | --- | --- | --- | --- |
| ROC-AUC | 0.746 ± 0.035 | 0.739 ± 0.031 | 0.746 ± 0.032 | 0.743 ± 0.032 | **0.772 ± 0.025** |
| PR-AUC | 0.127 ± 0.02 | 0.140 ± 0.03 | 0.141 ± 0.03 | 0.126 ± 0.03 | **0.152 ± 0.03** |
| Accuracy | **0.998 ± 0.0001** | **0.998 ± 0.0001** | **0.998 ± 0.0001** | 0.995 ± 0.009 | 0.993 ± 0.005 |
| Balanced accuracy | 0.511 ± 0.008 | 0.511 ± 0.011 | 0.51 ± 0.01 | 0.505 ± 0.008 | **0.558 ± 0.018** |
| Precision | 0.044 ± 0.032 | 0.041 ± 0.039 | 0.045 ± 0.052 | 0.027 ± 0.034 | **0.142 ± 0.045** |
| Recall | 0.022 ± 0.016 | 0.022 ± 0.022 | 0.02 ± 0.021 | 0.014 ± 0.017 | **0.12 ± 0.037** |
| F1 score | 0.028 ± 0.019 | 0.027 ± 0.025 | 0.027 ± 0.029 | 0.015 ± 0.019 | **0.117 ± 0.034** |
| MCC | 0.03 ± 0.021 | 0.029 ± 0.027 | 0.029 ± 0.032 | 0.017 ± 0.021 | **0.122 ± 0.035** |

**Additional file 1: Table S5** - Summary of the benchmarking results for the Phosphatase dataset. The best values for each metric in each dataset are highlighted in bold.

| **Metric** | **FC** | **LA** | **LDAM** | **EQ** | **WCE** |
| --- | --- | --- | --- | --- | --- |
| ROC-AUC | **0.830 ± 0.001** | **0.830 ± 0.01** | 0.825 ± 0.0008 | 0.821 ± 0.0003 | 0.814 ± 0.0005 |
| PR-AUC | 0.154 ± 0.002 | **0.162 ± 0.004** | 0.158 ± 0.004 | 0.123 ± 0.002 | 0.121 ± 0.001 |
| Accuracy | **0.992 ± 4E-4** | **0.992 ± 3E-4** | **0.992 ± 2E-4** | **0.992 ± 7E-4** | 0.989 ± 0.0005 |
| Balanced accuracy | 0.559 ± 0.006 | 0.563 ± 0.002 | 0.553 ± 0.002 | 0.531 ± 0.007 | **0.581 ± 0.004** |
| Precision | 0.455 ± 0.05 | 0.431 ± 0.06 | 0.567 ± 0.05 | **0.571 ± 0.01** | 0.356 ± 0.01 |
| Recall | 0.125 ± 0.01 | 0.135 ± 0.01 | 0.109 ± 0.03 | 0.085 ± 0.02 | **0.139 ± 0.006** |
| F1 score | 0.196 ± 0.01 | **0.206 ± 0.01** | 0.182 ± 0.02 | 0.148 ± 0.01 | 0.200 ± 0.003 |
| MCC | 0.177 ± 0.018 | **0.216 ± 0.011** | 0.212 ± 0.01 | 0.123 ± 0.006 | 0.189 ± 0.004 |

**Additional file 1: Table S6** - Summary of the benchmarking results for the NTPase dataset. The best values for each metric in each dataset are highlighted in bold.

| **Metric** | **FC** | **LA** | **LDAM** | **EQ** | **WCE** |
| --- | --- | --- | --- | --- | --- |
| ROC-AUC | 0.787 ± 0.01 | **0.852 ± 0.01** | 0.827 ± 0.02 | 0.764 ± 0.007 | 0.821 ± 0.01 |
| PR-AUC | **0.316 ± 0.002** | 0.308 ± 0.005 | 0.311 ± 0.001 | 0.208 ± 0.019 | 0.291 ± 0.001 |
| Accuracy | 0.945 ± 0.004 | 0.945 ± 0.0004 | **0.946 ± 0.005** | 0.899 ± 0.02 | 0.945 ± 0.001 |
| Balanced accuracy | 0.612 ± 0.007 | 0.61 ± 0.004 | 0.608 ± 0.008 | 0.553 ± 0.009 | **0.647 ± 0.007** |
| Precision | 0.417 ± 0.01 | 0.472 ± 0.01 | **0.488 ± 0.006** | 0.344 ± 0.04 | 0.381 ± 0.01 |
| Recall | 0.294 ± 0.005 | 0.267 ± 0.003 | 0.255 ± 0.005 | 0.250 ± 0.02 | 0.300 ± 0.007 |
| F1 score | **0.345 ± 0.004** | 0.341 ± 0.005 | 0.335 ± 0.003 | 0.289 ± 0.03 | 0.336 ± 0.003 |
| MCC | 0.278 ± 0.011 | **0.282 ± 0.006** | 0.279 ± 0.01 | 0.157 ± 0.018 | 0.271 ± 0.003 |

1. **Statistical analysis**

**Additional file 1: Table S7** – Significance levels for the Welch tests after Bonferroni correction for each dataset.

| **Dataset** | **Task number** | **α = 0.05** | **α = 0.01** |
| --- | --- | --- | --- |
| HIV | 1 | 0.05 | 0.01 |
| Tox21 | 12 | 0.004166 | 0.000833 |
| MUV | 17 | 0.002941 | 0.000588 |
| Phosphatase | 5 | 0.01 | 0.002 |
| NTPase | 6 | 0.008333 | 0.001666 |
| HTS | 1 | 0.05 | 0.01 |

**Additional file 1: Table S8** - P-values of the Welch tests (N = 50) for the HIV dataset against WCE.

| **Metric** | **FC** | **LA** | **LDAM** | **EQ** |
| --- | --- | --- | --- | --- |
| ROC-AUC | 8E-7 | 0.0007 | 1E-6 | 1 |
| PR-AUC | 0.007 | 0.81 | 0.05 | 1 |
| Accuracy | 2.92E-06 | 0.07 | 0.004 | 0.71 |
| Balanced accuracy | 0.97 | 0.94 | 0.78 | 1 |
| Precision | 8.27E-08 | 0.02 | 0.001 | 3.42E-07 |
| Recall | 0.98 | 0.95 | 0.81 | 1 |
| F1 score | 0.26 | 0.78 | 0.28 | 1 |
| MCC | 0.01 | 0.55 | 0.06 | 1 |

**Additional file 1: Table S9** - P-values of the Welch tests (N = 50) for the Tox21 dataset against WCE.

| **Metric** | **FC** | **LA** | **LDAM** | **EQ** |
| --- | --- | --- | --- | --- |
| ROC-AUC | 2E-11 | 9E-14 | 7E-11 | 0.99 |
| PR-AUC | 0.75 | 0.91 | 0.03 | 1 |
| Accuracy | 9.28E-06 | 2.71E-05 | 1.50E-09 | 0.86 |
| Balanced accuracy | 0.99 | 0.99 | 0.99 | 1 |
| Precision | 8.87E-06 | 8.12E-06 | 3.70E-10 | 1 |
| Recall | 1 | 1 | 0.99 | 1 |
| F1 score | 0.99 | 0.99 | 0.97 | 1 |
| MCC | 0.83 | 0.89 | 0.24 | 1 |

**Additional file 1: Table S10** - P-values of the Welch tests (N = 50) for the MUV dataset against WCE.

| **Metric** | **FC** | **LA** | **LDAM** | **EQ** |
| --- | --- | --- | --- | --- |
| ROC-AUC | 0.99 | 0.99 | 0.99 | 0.99 |
| PR-AUC | 1 | **1** | 1 | 1 |
| Accuracy | 2.45E-06 | 2.22E-06 | 2.16E-06 | 0.15675566 |
| Balanced accuracy | 1 | 1 | 1 | 1 |
| Precision | 1 | 1 | 1 | 1 |
| Recall | 1 | 1 | 1 | 1 |
| F1 score | 1 | 1 | 1 | 1 |
| MCC | 1 | 1 | 1 | 1 |

**Additional file 1: Table S11** - P-values of the Welch tests (N = 5) for the Phosphatase dataset against WCE.

| **Metric** | **FC** | **LA** | **LDAM** | **EQ** |
| --- | --- | --- | --- | --- |
| ROC-AUC | 1E-07 | 9E-6 | 2E-08 | 2E-08 |
| PR-AUC | 8.40E-08 | 4.03E-06 | 7.61E-06 | 0.02 |
| Accuracy | 0.0001 | 8E-5 | 0.001 | 0.0001 |
| Balanced accuracy | 0.99 | 0.99 | 0.99 | 0.99 |
| Precision | 0.005 | 0.02 | 0.0002 | 0.007 |
| Recall | 1 | 1 | 1 | 1 |
| F1 score | 1 | 0.2 | 1 | 1 |
| MCC | 0.90 | 0.001 | 0.002 | 0.99 |

**Additional file 1: Table S12** - P-values of the Welch tests (N = 5) for the NTPase dataset against WCE.

| **Metric** | **FC** | **LA** | **LDAM** | **EQ** |
| --- | --- | --- | --- | --- |
| ROC-AUC | 1 | 0.003 | 0.3 | 1 |
| PR-AUC | 8.06E-07 | 0.0003 | 1.69E-07 | 0.99 |
| Accuracy | 0.07 | 0.4 | 0.1 | 1 |
| Balanced accuracy | 0.99 | 0.99 | 0.99 | 0.99 |
| Precision | 0.001 | 0.000005 | 0.000001 | 1 |
| Recall | 1 | 1 | 1 | 1 |
| F1 score | 0.001 | 0.04 | 0.6 | 1 |
| MCC | 0.13 | 0.004 | 0.09 | 0.99 |

**Additional file 1: Table S13** – P-values of the Welch tests (N = 50) for the datasets from the MoleculeNet repository against the models from Arshadi et al.

| **Dataset** | **Baseline** | **FC** | **LA** | **EQ** | **LDAM** |
| --- | --- | --- | --- | --- | --- |
| HIV | SVM – Jiang | N.A. | N.A. | N.A. | 0.007 |
|  | GCN – Jiang | N.A. | N.A. | N.A. | 0.9921 |
| Tox21 | RF - Jiang | N.A. | 1 | N.A. | N.A. |
|  | AFP - Jiang | N.A. | 1 | N.A. | N.A. |
| MUV | SVM – Jiang | N.A. | N.A. | N.A. | 0.0003 |
|  | GCN - Jiang | N.A. | N.A. | N.A. | 2E-20 |

1. **Proprietary dataset analysis**

**Additional file 1: Table S14** - Summary of the benchmarking results for the HTS dataset. The best values for each metric in each dataset are highlighted in bold.

| **Metric** | **FC** | **LA** | **LDAM** | **EQ** | **WCE** |
| --- | --- | --- | --- | --- | --- |
| ROC-AUC | **0.925 ± 0.0004** | 0.923 ± 0.0005 | 0.921 ± 0.0005 | 0.873 ± 0.0007 | 0.915 ± 0.0004 |
| PR-AUC | **0.108 ± 0.001** | 0.107 ± 0.003 | 0.09 ± 0.001 | 0.024 ± 0.019 | 0.083 ± 0.002 |
| Accuracy | **0.992 ± 0.002** | 0.987 ± 0.002 | 0.989 ± 0.001 | 0.51 ± 0.412 | 0.982 ± 0.002 |
| Balanced accuracy | 0.648 ± 0.016 | 0.699 ± 0.012 | 0.664 ± 0.004 | 0.527 ± 0.055 | **0.709 ± 0.008** |
| Precision | **0.097 ± 0.019** | 0.072 ± 0.01 | 0.076 ± 0.005 | 0.011 ± 0.018 | 0.055 ± 0.003 |
| Recall | 0.303 ± 0.033 | 0.41 ± 0.026 | 0.337 ± 0.01 | **0.544 ± 0.388** | 0.435 ± 0.019 |
| F1 score | **0.144 ± 0.017** | 0.121 ± 0.013 | 0.123 ± 0.006 | 0.019 ± 0.031 | 0.098 ± 0.004 |
| MCC | **0.166 ± 0.008** | **0.166 ± 0.007** | 0.156 ± 0.004 | 0.023 ± 0.042 | 0.15 ± 0.001 |

**Additional file 1: Table S15** - P-values of the Welch tests (N = 5) for the HTS dataset against WCE.

| **Metric** | **FC** | **LA** | **LDAM** | **EQ** |
| --- | --- | --- | --- | --- |
| ROC-AUC | 8E-11 | 1E-09 | 1E-08 | 1 |
| PR-AUC | 1.77E-07 | 3.04E-07 | 0.0006 | 0.99 |
| Accuracy | 1.53E-05 | 0.005 | 7.61E-05 | 0.96 |
| Balanced accuracy | 0.99 | 0.92 | 0.99 | 0.99 |
| Precision | 0.004 | 0.009 | 6.80E-05 | 0.99 |
| Recall | 0.99 | 0.93 | 0.99 | 0.28 |
| F1 score | 0.001 | 0.006 | 5.55E-05 | 0.99 |
| MCC | 0.005 | 0.003 | 0.01 | 0.99 |

1. **Convergence speed analysis**

**Additional file 1: Table S16** – Training times (s) for each loss function with optimal hyperparameters for the HIV dataset.

| **Run ID** | **WCE** | **FC** | **LA** | **EQ** | **LDAM** |
| --- | --- | --- | --- | --- | --- |
| 1 | 45.3 | 22.4 | 4.1 | 22.5 | 5.9 |
| 2 | 42.9 | 22.4 | 4.2 | 17.9 | 6.0 |
| 3 | 43.3 | 23.0 | 4.5 | 56.8 | 6.2 |
| 4 | 42.9 | 22.3 | 4.0 | 53.2 | 6.4 |
| 5 | 45.3 | 21.8 | 3.9 | 55.1 | 6.1 |
| 6 | 61.1 | 19.8 | 10.4 | 6.6 | 6.1 |
| 7 | 60.5 | 28.6 | 19.1 | 6.1 | 5.4 |
| 8 | 64.2 | 28.3 | 19.1 | 7.8 | 5.5 |
| 9 | 61.7 | 28.3 | 19.9 | 7.3 | 5.6 |
| 10 | 60.0 | 29.2 | 19.4 | 7.9 | 5.8 |
| 11 | 74.1 | 8.5 | 18.7 | 116.3 | 9.8 |
| 12 | 70.9 | 9.1 | 20.5 | 67.1 | 9.2 |
| 13 | 73.9 | 9.3 | 20.0 | 74.0 | 9.6 |
| 14 | 71.1 | 8.9 | 19.9 | 115.4 | 9.6 |
| 15 | 72.9 | 8.9 | 20.1 | 107.7 | 9.3 |
| Average | 59.3 | 19.4 | 13.9 | 48.1 | 7.1 |

**Additional file 1: Table S17** – Boosting iterations for each loss function with optimal hyperparameters for the HIV dataset.

| **Run ID** | **WCE** | **FC** | **LA** | **EQ** | **LDAM** |
| --- | --- | --- | --- | --- | --- |
| 1 | 4580 | 772 | 163 | 1823 | 290 |
| 2 | 4101 | 772 | 171 | 1823 | 290 |
| 3 | 5000 | 772 | 171 | 5000 | 290 |
| 4 | 5000 | 772 | 171 | 5000 | 290 |
| 5 | 5000 | 772 | 171 | 5000 | 290 |
| 6 | 5000 | 689 | 380 | 265 | 461 |
| 7 | 5000 | 1019 | 774 | 265 | 415 |
| 8 | 5000 | 1019 | 774 | 265 | 415 |
| 9 | 5000 | 1019 | 774 | 265 | 415 |
| 10 | 5000 | 1019 | 774 | 265 | 415 |
| 11 | 5000 | 425 | 1269 | 5000 | 716 |
| 12 | 5000 | 444 | 1364 | 5000 | 716 |
| 13 | 5000 | 444 | 1364 | 5000 | 716 |
| 14 | 5000 | 443 | 1364 | 5000 | 716 |
| 15 | 5000 | 443 | 1364 | 5000 | 716 |
| Average | 4912.1 | 721.6 | 736.5 | 2998.1 | 476.7 |

1. **References**

(1) Feng, Y.; Zhou, M.; Tong, X. Imbalanced Classification: A Paradigm-Based Review. arXiv June 30, 2021.

(2) Chicco, D.; Jurman, G. The Advantages of the Matthews Correlation Coefficient (MCC) over F1 Score and Accuracy in Binary Classification Evaluation. *BMC Genomics* **2020**, *21* (1), 6. https://doi.org/10.1186/s12864-019-6413-7.

(3) Jiang, D.; Wu, Z.; Hsieh, C.-Y.; Chen, G.; Liao, B.; Wang, Z.; Shen, C.; Cao, D.; Wu, J.; Hou, T. Could Graph Neural Networks Learn Better Molecular Representation for Drug Discovery? A Comparison Study of Descriptor-Based and Graph-Based Models. *J. Cheminformatics* **2021**, *13* (1), 12. https://doi.org/10.1186/s13321-020-00479-8.

(4) Keshavarzi Arshadi, A.; Salem, M.; Firouzbakht, A.; Yuan, J. S. MolData, a Molecular Benchmark for Disease and Target Based Machine Learning. *J. Cheminformatics* **2022**, *14* (1), 10. https://doi.org/10.1186/s13321-022-00590-y.

(5) Fu, G.; Yi, L.; Pan, J. Tuning Model Parameters in Class‐imbalanced Learning with Precision‐recall Curve. *Biom. J.* **2019**, *61* (3), 652–664. https://doi.org/10.1002/bimj.201800148.
